# An ABA-increased interaction of the PYL6 ABA receptor with MYC2 Transcription Factor: A putative link of ABA and JA signaling

**DOI:** 10.1038/srep28941

**Published:** 2016-06-30

**Authors:** Fernando Aleman, Junshi Yazaki, Melissa Lee, Yohei Takahashi, Alice Y. Kim, Zixing Li, Toshinori Kinoshita, Joseph R. Ecker, Julian I. Schroeder

**Affiliations:** 1 Division of Biological Sciences, Cell and Developmental Biology Section, University of California San Diego, La Jolla, California, USA; 2 Plant Biology Laboratory, Genomic Analysis Laboratory, The Salk Institute for Biological Studies, La Jolla, California, 92037 USA; 3 Division of Biological Science, Graduate School of Science, Nagoya University, Nagoya 464-8602, Japan; 4 Institute of Transformative Bio-Molecules (WPI-ITbM), Nagoya University, Nagoya 464-8602, Japan; 5 Howard Hughes Medical Institute, The Salk Institute for Biological Studies, La Jolla, California, 92037 USA

## Abstract

Abscisic acid (ABA) is a plant hormone that mediates abiotic stress tolerance and regulates growth and development. ABA binds to members of the PYL/RCAR ABA receptor family that initiate signal transduction inhibiting type 2C protein phosphatases. Although crosstalk between ABA and the hormone Jasmonic Acid (JA) has been shown, the molecular entities that mediate this interaction have yet to be fully elucidated. We report a link between ABA and JA signaling through a direct interaction of the ABA receptor PYL6 (RCAR9) with the basic helix-loop-helix transcription factor MYC2. PYL6 and MYC2 interact in yeast two hybrid assays and the interaction is enhanced in the presence of ABA. PYL6 and MYC2 interact *in planta* based on bimolecular fluorescence complementation and co-immunoprecipitation of the proteins. Furthermore, PYL6 was able to modify transcription driven by MYC2 using JAZ6 and JAZ8 DNA promoter elements in yeast one hybrid assays. Finally, *pyl6* T-DNA mutant plants show an increased sensitivity to the addition of JA along with ABA in cotyledon expansion experiments. Overall, the present study identifies a direct mechanism for transcriptional modulation mediated by an ABA receptor different from the core ABA signaling pathway, and a putative mechanistic link connecting ABA and JA signaling pathways.

Environmental stresses affect plant growth and crop yield worldwide, and consequently, threaten food supply for a growing population. To cope with the deleterious effects of abiotic stresses, plants respond with the synthesis of hormones, which mediate changes in gene expression^1^. The plant hormone abscisic acid (ABA) is a major player in abiotic stress tolerance, and is involved in regulating plant growth and development^2^,^3^,^4^,^5^. The PYL/RCAR ABA receptor family comprises 14 members in the reference plant *Arabidopsis thaliana*, and they have been subdivided into 3 subgroups based on sequence similarity^6^,^7^. Members of the subgroup III (PYR1, PYL1, PYL2 and PYL3) have a higher probability of forming receptor dimers^8^,^9^,^10^,^11^,^12^,^13^. This subgroup of ABA receptors displays a lower ABA affinity compared with members of the family that have a higher probability of residing in the monomeric form^11^,^12^,^14^.

The receptors interact with the clade A PP2C protein phosphatases^6^,^7^,^15^,^16^,^17^. Some ABA receptors are able to interact in yeast with some PP2Cs even in the absence of ABA^6^,^7^,^18^. In the presence of ABA, PP2C activity is inhibited. However, the interaction observed between the ABA receptors and PP2Cs in yeast, in the absence of ABA, does not always result in the inhibition of PP2C protein phosphatase activity *in vitro*^6^,^7^,^12^,^19^,^20^,^21^.

No longer inhibited by the PP2C protein phosphatases in the presence of ABA, SnRK2 Serine/Threonine protein kinases are able to phosphorylate diverse downstream targets that include the ion channels SLAC1 or KAT1 22,23,24,25,26, and transcription factors (TFs) such as ABI5 or AREB1 4. Other ABA signaling pathways that diverge from this traditional signaling module remain to be identified and the molecular entities that mediate crosstalk of ABA signal transduction with other hormones^27^ remain to a large degree unknown.

PYL6/RCAR9 belongs to the subgroup II of ABA receptors along with PYL4/RCAR10 and PYL5/RCAR8 6,7,16. Similar to other ‘monomeric’ ABA receptors, PYL6/RCAR9 displays high-affinity for ABA binding^11^. This subgroup of ABA receptors is up- or down-regulated at the mRNA level by JA^28^. In addition, Arabidopsis *pyl4* and *pyl5* T-DNA single mutants have been shown to be more sensitive to JA in shoot growth assays^28^. However, the mechanisms underlying the interaction of PYR/RCAR receptors with jasmonate signaling remain unknown.

The Arabidopsis genome comprises over 2000 TFs harboring some of the largest protein families^29^. MYC2 is a basic helix-loop-helix (bHLH) leucine zipper TF also known as *jin1*/*jai1* 30. MYC2 was first isolated in a screening for proteins that bind to the promoter of RD22, which was one of the first Arabidopsis genes described to be ABA-induced^31^. MYC2 was further characterized as a positive regulator of ABA signaling through knock-out and overexpression mutants^32^. Then, MYC2 emerged as a central regulator in JA signaling^30^,^33^,^34^,^35^,^36^. But the genes and mechanisms involved in ABA-JA crosstalk have not yet been identified.

In this study, we have identified and characterized a key interaction between the ABA receptor PYL6 and the TF MYC2. Both proteins interact in yeast two hybrid assays and the interaction is further increased in the presence of ABA. Furthermore, PYL6-MYC2 interaction was confirmed *in planta* through co-immunoprecipitation of the proteins, and BiFC analyses suggest a nuclear localization of the interaction. Additionally, mutant plants lacking a functional PYL6 showed altered sensitivity to ABA compared to control plants in a cotyledon greening assay, and the effect was further increased when JA was added with ABA. Finally, PYL6 was able to modify the transcriptional activity of MYC2 in a yeast one hybrid assays using promoter elements of the JAZ6 and JAZ8 genes containing the G-box sequence.

## Results

### PYL6 can interact with MYC2 in yeast two hybrid assays

**Figure 1:**
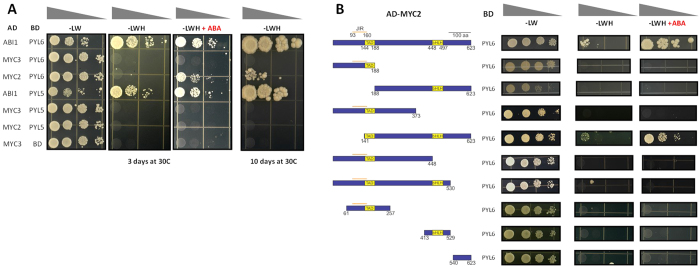
PYL6 interacts with MYC2 in yeast and the interaction is enhanced by ABA. (**A)** Drop test assays show growth of yeast after 3 days in the presence of 10 μM ABA for the PYL6-MYC2 interaction pair and the positive control PYL6-ABI1 (presence of ABA (+ABA) is indicated on top of the panel; number of days of plate growth is indicated on the bottom). No interaction was found with the closest homologues of MYC2 (MYC3-PYL6) and PYL6 (MYC2-PYL5), suggesting a specific interaction. After 10 days of growth (right panel) PYL6-MYC2 interaction can be observed in the absence of ABA. **(B)** Using truncated versions of MYC2, the required region for MYC2-PYL6 interaction was mapped. Left side shows a cartoon with the different MYC2 truncated versions investigated in Y2H experiments. Only the truncated version MYC2 141–623 (amino acid numbers) was able to interact with PYL6. The enhancement by ABA was also observed for the truncated MYC2 (141–623). Negative controls with empty vector are shown in Figure S1.

We performed a study of protein-protein interactions using a high-density protein array, where 12,000 Arabidopsis proteins were synthetized on glass slides and tested for interactions with 38 Transcription Factors (TF) proteins that function in diverse plant hormone regulatory pathways (Yazaki *et al*., Mapping transcription factor interactome networks using HaloTag protein arrays, PNAS in press). A putative interaction between the ABA receptor PYL6 with the TF MYC2 was further investigated by yeast two hybrid analyses (Fig. 1A). Interestingly, when ABA was present in triple drop-out media, the interaction between PYL6 and MYC2 was enhanced (Fig. 1A). To test for the specificity of this interaction in yeast, we investigated the closest homologue of PYL6, PYL5, and the closest homologue of MYC2, MYC3, and did not observe PYL5 interaction with MYC2 or MYC3, nor PYL6 interaction with MYC3 (Fig. 1A). As a positive control, ABI1 was used and interactions of PYL6 and PYL5 with ABI1 were observed in the presence and absence of ABA (Fig. 1A)^12^,^16^.

We further pursued yeast two hybrid analyses to gain insight into the regions of MYC2 necessary for the interaction with PYL6 (Fig. 1B). A truncated MYC2 (141–623) lacking the first 140 amino acids was able to interact with PYL6 (Fig. 1B; Figure S1). ABA also enhanced the interaction of PYL6 with truncated MYC2 (141–623), as it did with full length MYC2 (Fig. 1B).

### PYL6-MYC2 interaction also occurs in planta

**Figure 2:**
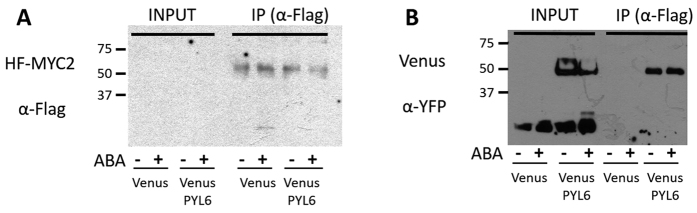
MYC2 and PYL6 co-immunoprecipitate *in planta*. *N. benthamiana* leaves were co-infiltrated with Flag-MYC2 and Venus-PYL6 or Venus alone. Protein extractions from leaves were split in 2 halves and either ABA-treated or mock-treated (indicated at the bottom of the panels). Immunoprecipitation was performed with anti-Flag beads against MYC2 (IP lanes indicated on the top right of each panel). (**A)** Left panel shows MYC2 detection with anti-Flag antibody suggesting low MYC2 expression levels before the purification. (**B)** Venus or Venus-PYL6 protein were detected using an anti-YFP antibody, and both can be observed in the input samples. Venus-PYL6, and not Venus alone, was co-immunoprecipitated with MYC2 (IP lanes).

Whether PYL6-MYC2 interaction occurs *in planta* was investigated by co-immunoprecipitation assays in *N. benthamiana*. HF-MYC2 was co-expressed in *N. benthamiana* leaves with Venus-PYL6 or Venus alone. An anti-Flag antibody was used to pull down MYC2 (Fig. 2A). The protein expression levels of MYC2 were not sufficient to be detected in the input samples of the Western blot, but became detectable after immunoprecipitation suggesting low MYC2 protein expression (Fig. 2A; IP lanes). An anti-YFP antibody was used to detect either Venus-PYL6 (~50 kDa) or Venus as control (~25 kDa; Fig. 2B). Both were detected in the input samples, while only Venus-PYL6 and not Venus was detected after MYC2 purification (Fig. 2B). The presence or absence of ABA did not have an observable effect under the imposed conditions (see Discussion).

### PYL6-MYC2 interaction occurs in the nucleus

**Figure 3:**
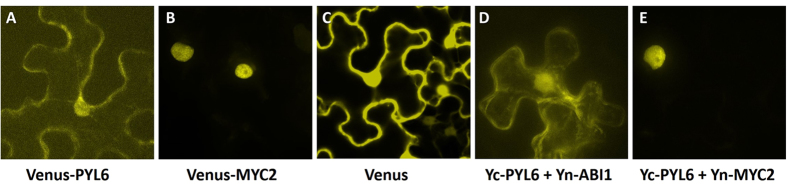
PYL6 interacts with MYC2 in the nucleus. (**A,B**) Subcellular localization of (**A**) PYL6 and (**B**) MYC2 tagged with Venus fluorescence protein. Venus-PYL6 was localized in the cytoplasm and nucleus and Venus-MYC2 was localized exclusively in the nucleus. (**C,D)** BiFC experiments show that (**C**) Yc-PYL6 interacts with Yn-ABI1 in the cytoplasm and nucleus of *N. benthamiana* leaves cells while (**D**) the interaction of Yc-PYL6 with Yn-MYC2 was only observed in the nucleus.

The subcellular localization of Venus-PYL6 and Venus-MYC2 along with the subcellular localization of any PYL6-MYC2 interaction were investigated (Fig. 3). Venus-PYL6 was localized in the cytoplasm and nucleus as are other ABA receptors^6^,^7^,^15^,^37^,^38^ (Fig. 3A). Venus-MYC2 was targeted only to the nucleus (Fig. 3B), while Venus only localized to the cytoplasm and nucleus (Fig. 3C). In control BiFC experiments, the N-terminal half of YFP fused to ABI1 (YFPc-ABI1) was able to interact with the C-terminal half of YFP fused to PYL6 (YFPn-PYL6) in the cytoplasm and the nucleus (Fig. 3D). An interaction between YFPn-PYL6 and YFPc-MYC2 was detected, and only occurred in the nucleus (Fig. 3E).

### pyl6 T-DNA mutants are more sensitive to JA + ABA

In order to test the putative biological relevance of the interaction of PYL6 and MYC2, Arabidopsis mutants were isolated. We tested ~200 T1 35S::YFP-PYL6 overexpression lines selected on hygromycin plates coming from 2 independent transformations of 6 T0 plants. 15 of those hygromycin-resistant T1 plants were selected to obtain T2 seeds in case homozygosity of the 35S::YFP-PYL6 transgene would improve expression Only one seedling showed fluorescence in the T2 generation and that plant had severe growth and developmental defects and died without producing offspring. These data suggest a unique or possible detrimental function of PYL6 compared to other successfully over-expressed ABA receptors^6^,^7^,^38^,^39^,^40^.

**Figure 4:**
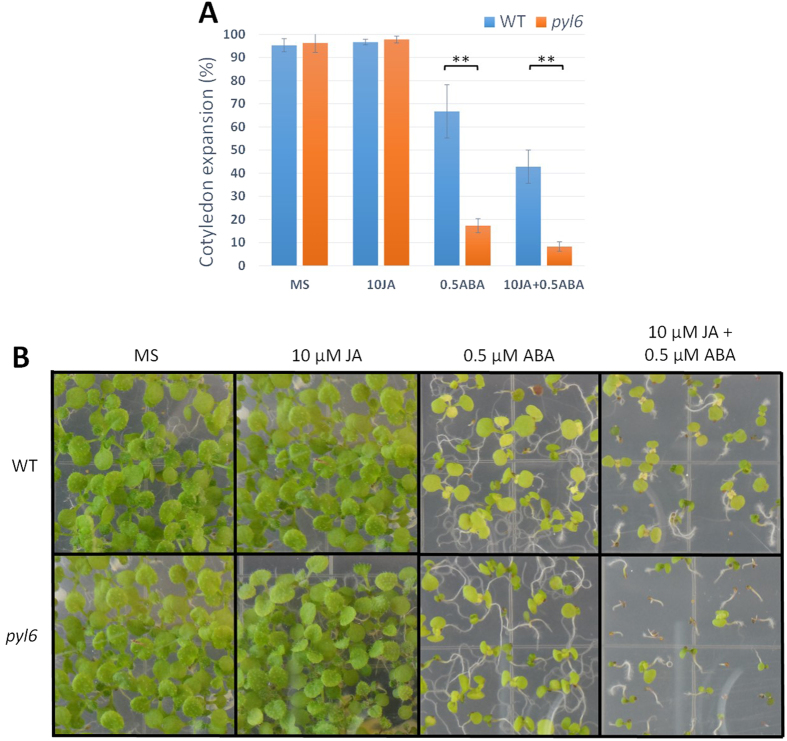
*pyl6* mutant plants are more sensitive to JA + ABA. *pyl6* seedlings are more sensitive to ABA and to the combination of ABA and JA than WT (Col-0). (**A)** 5 days after sowing expanded and green cotyledons were quantified per number of plants analyzed (%). Data are represented as mean ± SD and **Indicates *p*-value <0.01 after a two-tailed t-test. Images of the seedlings at this stage can be found in Supplementary Figure S4. (**B)** In 11 day-old seedlings, the synergistic action of JA over the ABA effect was enhanced in *pyl6* mutants compared to WT. Right panels show 0.5 μM ABA plus 10 μM Me-JA.

A *pyl6* T-DNA insertion mutant was isolated and sequenced. The T-DNA is inserted in the 3′ terminus of the PYL6 ORF, truncating the last 11 amino acids of the last α-helix and introducing an asparagine before a stop codon. Thus, a putative truncated version of the PYL6 protein with an asparagine residue added at the N-terminus was cloned and analyzed in yeast two hybrid assays with MYC2 and ABI1 (Figure S2). This potential truncated PYL6∆C protein that may be encoded in the *pyl6* mutant did not interact with MYC2 in the presence or absence of ABA (Figure S2). Furthermore, ABI1 only interacted with this PYL6∆C truncated version in the presence of ABA and not in the absence of ABA (Figure S2). The C-terminal α–helix has several critical residues that affect ABA binding^10^ and a PYL4 A194T substitution located in this C-terminal α-helix also modified the interaction pattern and ABA affinity with PP2CA but not with HAB1 in Y2H experiments^39^. Using the adapter ligation-mediated PCR method^41^ with whole genomic DNA of *pyl6* mutant plants we could only amplify a fragment corresponding to PYL6 from the Arabidopsis genome, strongly suggesting the presence of only one T-DNA insertion. Therefore, the *pyl6* T-DNA line was selected for further germination, post-germination and root growth analyses. We found no germination differences, as defined by radicle emergence, between Col-0 and *pyl6* in control MS media or media supplemented with 0.5 μM ABA, 10 μM JA nor a combination of ABA and JA (Figure S3). However, after 5 days of growth, *pyl6* seedlings showed an enhanced sensitivity to ABA and to ABA + JA (Fig. 4A; Figure S4). While 66% of Col-0 seeds were able to develop green cotyledons in the presence of ABA, only 17% of *pyl6* seeds did. When both ABA and JA were added only 45% of Col-0 and 8% of *pyl6* plants developed green cotyledons (Fig. 4A). This accounts for a 35% and 52% further reduction due to the addition of JA for Col-0 and *pyl6* respectively. After 11 days of growth, only small differences were found for Col-0 between ABA and ABA + JA treatments, however *pyl6* mutant plants showed an increased sensitivity to ABA + JA compared with ABA alone (Fig. 4B).

### PYL6 modifies the transcriptional activity of MYC2 in yeast

**Figure 5:**
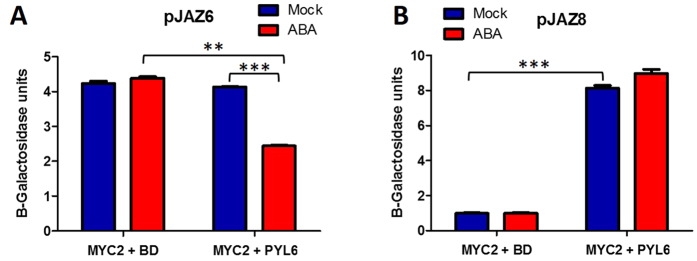
PYL6 modifies the transcriptional activity of MYC2 in yeast one hybrid (Y1H) analyses. **(A)** MYC2 alone induced transcription driven by JAZ6 promoter. The presence of PYL6 and ABA reduced MYC2 transcriptional activity. (**B)** MYC2 alone did not induce transcription driven by JAZ8 promoter fragment. PYL6 strongly activates MYC2 transcription activity with a JAZ8 promoter fragment. Error bars indicate standard deviation of at least 3 independent transformants. ***Indicate the results of a two-tailed t-test with *p* values ≤0.001 and **Represent *p* value ≤0.01. BD indicates the empty vector where PYL6 was cloned.

We further investigated the effects of the PYL6-MYC2 interaction on transcriptional control. We used a yeast one hybrid approach using a JA-induced DNA fragment from the JAZ8 promoter and an additional JA-induced fragment from the JAZ6 promoter^42^. Both promoters contain the G-box sequence to which MYC2 binds^43^. MYC2 alone activated transcription driven by the JAZ6 promoter in the presence or absence of ABA (Fig. 5A left columns). The addition of PYL6 in the absence of ABA had no effect, but interestingly the addition of PYL6 and ABA reduced the transcriptional activity by 41% (Fig. 5A; right columns). MYC2 alone did not induce above background the activity of the JAZ8 promoter (Fig. 5B, left columns). However, interestingly the addition of PYL6 caused a sharp increase in transcription of the *β*-galactosidase (Fig. 5B, right columns). ABA did not produce a significant effect probably due to the saturation of the system. Other promoter fragments, RD22 32 and TPS21 44, without a G-box sequence, did not produce any MYC2 transcriptional activity under our experimental conditions.

### MYC2 does not affect the ABI1 inhibition by PYL6

In additional experiments, we investigated whether the inhibitory effect of PYL6 on the protein phosphatase activity of ABI1 was impaired by the presence of MYC2 *in vitro*. Recombinant proteins were used in a protein phosphatase activity assay using 60 nM ABI1 and 10 μM ABA (Figure S5). ABI1 protein phosphatase activity in the presence of ABA was not altered after the addition of 90 nM of MYC2 protein or the same concentration of BSA. In contrast, 90 nM of PYL6 in the presence of ABA inhibited 50% of the PP2C activity. This inhibition by PYL6 was not significantly altered after the addition of 90 nM of MYC2 or BSA (Figure S5).

## Discussion

Signal transduction pathways are based on protein-protein interactions and protein-ligand interactions that transduce information to produce specific cellular outputs. Moreover, environmental and developmental signals often require changes in gene expression to adapt to abiotic stress conditions^45^,^46^,^47^. Therefore, a key determinant of a cell’s ability to modulate environmental and developmental cues lies in the interaction of TFs with other proteins that regulate their transcriptional activity or stability^29^,^48^. High-throughput protein arrays offer a valuable resource to probe the protein interactome, and like all high-throughput methods, require validation of the protein interactions found^49^,^50^,^51^,^52^. Here we report a direct interaction of the Arabidopsis ABA receptor PYL6 and the bHLH transcription factor MYC2. We observed that in yeast two hybrid experiments, PYL6 can interact with MYC2, and this interaction was enhanced when ABA was present in the media (Fig. 1A). We further tested the closest homologues to PYL6 and MYC2: PYL5 and MYC3, respectively. PYL6 did not interact with MYC3, nor did MYC2 with PYL5. These results suggest a molecular specificity of the MYC2-PYL6 interaction.

Furthermore, using an array of truncated versions of MYC2, we determined that the only mutated MYC2 version (amino acids 141-623) that interacted with PYL6 also showed an ABA-enhanced interaction in yeast (Fig. 1B). Previously Y2H assays have been used to identify ABA receptors^6^,^7^, to isolate ABA transporters^53^, to screen for ABA analogues^14^, and Y2H has been used to define the interaction of specific PP2C-ABA receptor pairs^15^,^18^,^37^ including in tomato^21^ and grape^54^.

In Y2H experiments, MYC2 residues 93-160 have been shown to be sufficient for the interaction with most JAZ proteins^55^, and also with some MYB TFs that regulate glucosinolate biosynthesis^56^. However, the residues 1–373 of MYC2, did not interact with PYL6 (Fig. 1B), and the complete JAZ interaction domain (amino acids 93–160) was not required for the interaction of MYC2 with PYL6 in Y2H experiments (amino acids 141–623; Fig. 1B; Figure S2). The interaction between MYC2 and PYL6 required almost the full MYC2 protein suggesting that different motifs of MYC2 or a proper folding are required for the PYL6-MYC2 interaction. However, the MYC2 region required for the interaction between MEDIATOR 25 (MED25) and MYC2 (residues 149–188) corresponding to the Transcriptional Activation Domain^42^ was included in the PYL6-MYC2 (141–623) interaction constructs. These data indicated that PYL6 may have the capacity to modify the transcriptional activity of MYC2 upon protein-protein interaction, and that ABA might affect gene expression driven by MYC2 through PYL6.

We further confirmed the PYL6-MYC2 interaction through *in planta* co-immunoprecipitation. After MYC2 purification with anti-Flag magnetic beads, Venus-PYL6 but not Venus alone was co-purified with MYC2 (Fig. 2B). Leaf expression levels of MYC2 were low and the protein could only be visualized after immunoprecipitation (Fig. 2A). A difference in the PYL6-MYC2 co-immunoprecipitation with or without ABA was not observed. With the dynamic range of detection of Western blots, it is difficult to account for differences in interaction affinities. Moreover, the basal ABA concentrations in Agrobacterium transformed *N. benthamiana* leaves may be elevated, impairing the ability to detect ABA-dependent differences for high-affinity ABA-binding proteins such as PYL6 and other monomeric ABA receptors^11^,^12^.

Most ABA-regulated genes contain ABA-responsive elements (ABRE; PyACGTGG/TC) in their promoters^57^,^58^,^59^. The G-box sequence (CACGTG) is included in this *cis*-element which has been associated with a wide variety of gene expression changes in plants^60^,^61^,^62^. Since MYC2 binds the G-box sequence^33^,^43^,^55^, we investigated whether MYC2-dependent transcriptional activity may be modulated by the PYL6 receptor (Fig. 5). JAZ6 has two G-box sequences in its promoter, and JAZ8 has one close to the ATG^42^. MYC2 alone was transcriptionally active when using the JAZ6 promoter fragment (Fig. 5A; left two columns). PYL6 addition by its own did not have an effect, however, addition of PYL6 and ABA inhibited MYC2 activity driven by the JAZ6 promoter. On the other hand, using a JAZ8 promoter fragment, MYC2 alone could not activate transcription, but the addition of PYL6 greatly enhanced MYC2 activity (Fig. 5B). *In planta* gene expression analysis showed that Col-0 reduced *JAZ6* expression after ABA treatment which correlates with the yeast observation, and the effect on *pyl6* mutants was not evident *in planta* suggesting additional regulatory pathways may play a role *in vivo* (Figure S6). In addition, ABA enhances *JAZ8* expression in Col-0 plants, and this enhancement was reduced in *pyl6* mutants which also correlates with the transcriptional activity of MYC2 observed in yeast one hybrid assays (Figure S6).

**Figure 6:**
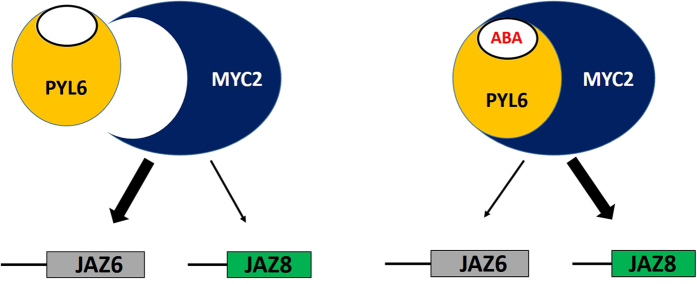
Proposed working model. Thick arrows indicate active transcription and dashed arrows weak transcription. Left: In the absence of ABA, PYL6 weakly interacts with MYC2, allowing the TF to bind a set of gene promoters such as JAZ6. When ABA is present, PYL6 strongly binds to MYC2 modifying its transcriptional activity, promoting the expression of JAZ8, whereas transcription via pJAZ6 is reduced. We propose that PYL6 acts as a transcriptional modulator upon interaction with MYC2.

JAZ promoter activity analyses suggest that PYL6 may modify the affinity of MYC2 for different promoters which ultimately may control gene expression changes in plants in response to ABA (Fig. 6). Many TFs have been shown to activate or repress gene expression depending on posttranslational modifications or interaction partners (e.g. ABI4 63). MYC2 has been postulated as a master regulator that modulates many signals^36^. For example, DELLA proteins, the main repressors of GA signaling, have been shown to repress JAZ proteins, which function in inhibiting MYC2 and JA-signaling, thus releasing and activating MYC2 64. Ethylene signaling has also been linked to MYC2 through interaction with EIN3^65^. Likewise the circadian-clock component Time for Coffee (TIC) interacts with MYC2 and prevents MYC2 protein accumulation^66^. Dark and shade also prevent MYC2 accumulation, while light and JA stabilize MYC2 protein^67^. Genetic experiments have also shown that MYC2 represses photomorphogenesis along with SPA1 68. All of these links among different signal transduction pathways require a versatile TF that activates or represses gene expression according to the target gene and a given stimulus^30^,^32^,^69^,^70^,^71^. However, the mechanisms have remained unclear how MYC2 functions as a positive regulator of ABA signaling^32^. The present findings provide a direct link between ABA signaling and MYC2, and indicate that the ABA receptor PYL6 could modulate MYC2 activity, supporting a direct pathway for ABA-induced gene expression control.

ABA and JA appear to function synergistically or antagonistically depending on the developmental stage, tissue type and response^70^,^72^,^73^,^74^. It has been reported that ABA and JA have synergistic effects on seed germination, and seedling cotyledon expansion and establishment^71^. Our results support this model (Fig. 4; Figures S3 and S4). Furthermore, *pyl4* and *pyl5* mutant plants, the two other ABA receptors from the same subgroup as PYL6, were described as hypersensitive to JA treatment, and the expression levels of this subgroup of ABA receptors change upon JA treatment^28^. We further identified a specific JA phenotype in the presence of ABA. The effect of JA with ABA on cotyledon greening was more pronounced in *pyl6* mutant plants (Fig. 4).

The putative truncated PYL6 protein that may be expressed in the *pyl6* T-DNA insertion mutant, did not interact with MYC2, but did show an ABA-induced ABI1 interaction (Figure S2). This indicates that in *pyl6* mutant plants, there may be a truncated PYL6 protein with an altered response to ABA. With MYC2 functioning as a positive regulator of ABA signaling^32^, the absence of PYL6-MYC2 interaction in the T-DNA mutant seedlings in the presence of ABA, may impair a PYL6-mediated direct regulation of specific MYC2 targets. This hypothesis will require future investigation. The direct interaction of the ABA receptor PYL6 with MYC2 characterized here may represent an ABA-receptor pathway with direct influence on TF activity.

There are few examples of reported ABA-receptor interactions with proteins different from clade A PP2Cs. High-throughput studies indicated some putative interactions of ABA receptors with other partners apart from the core signaling through PP2Cs^75^. Truncated PYL4 (RCAR10) and PYL9 (RCAR1), and full-length PYL8 (RCAR3) were able to interact, in an ABA-independent manner, with DDA1 to target PYL8 for ubiquitination and degradation^76^. Other TFs, MYB44 and MYB77, interact with the ABA receptor PYL8 77,78. MYB44 interacts with PYL8 in the absence of ABA to regulate leaf senescence^77^, and MYB77 and MYB44 interacted in yeast with PYL8 both in the absence or presence of ABA and the interaction was correlated with regulation of lateral root growth^78^. Conversely, tandem affinity purifications of PYL8 (RCAR3) in Arabidopsis cell cultures only identified interactors from clade A PP2Cs, but not any TF^79^. It was also reported that PYL8 could modify the transcriptional activity of MYB77 using the auxin reporter IAA19 in Arabidopsis protoplasts, and this modification of the activity was ABA-enhanced^78^. On the other hand, it was reported previously that PYL8 on its own can induce expression changes in Arabidopsis protoplasts through ABI1 37. To our knowledge, only two full-length ABA receptors, PYL8 and PYL6 as shown here, have been found to interact with transcription factors.

In summary, we report here the interaction of an ABA receptor PYL6 with the transcription factor MYC2 that provides a possible mechanistic link between ABA signaling and JA signaling. PYL6 and MYC2 interact in yeast and *in planta*, and we speculate that upon ABA binding to PYL6, MYC2 promoter affinity is changed to modulate gene expression changes during adaptation to environmental changes (Fig. 6). Further research will be needed to determine to which degree the PYL6-MYC2 interaction contributes to the regulation of biotic responses.

## Materials and Methods

### Yeast Two Hybrid (Y2H)

The *PYL6* cDNA was cloned in frame by USER cloning^80^ in the pGBT9.BS vector backbone (BD-PYL6), and *MYC2* cDNA was cloned in frame in pGAD.GH vector backbone (AD-MYC2). Plasmids were co-transformed into the yeast strain PJ69-4A^81^ and transformants were selected on SD double dropout media without Leucine and Tryptophan (SD-LW). Drop tests were done with 5 μL of 1/10 dilutions starting at OD_600_ of 1 until 0.001 in media without Leucine, Tryptophan and Histidine (SD-LWH) with the addition of 2.5 mM 3-Amino-1,2,4-triazole (3-AT). When indicated (±) ABA was added to a final concentration of 10 μM. Plates were grown at 30 °C for 10 days and photos were taken at days 3, 5, and 10.

### Bimolecular Fluorescence Complementation (BiFC)

The *PYL6* cDNA was cloned in frame by USER cloning^80^ in pSPYCE(MR) vector backbone (Yc-PYL6)^82^, and MYC2 in pSPYNE(R)173 (Yn-MYC2). These constructs were transformed into the agrobacterium GV3101 strain by electroporation. Agrobacteria were grown at 28 °C overnight along with the p19 strain^83^, and they were centrifuged at 4,000xg for 15 min. Cell pellets were resuspended with infiltration buffer (10 mM MES, 10 mM MgCl_2_ and 0.15 mM acetosyringone), and were adjusted to OD_600_ = 0.5, except for p19 that was adjusted at OD_600_ = 0.3. Cell suspensions were kept at RT for 2 hours and then infiltrated into *N. benthamiana* leaves and incubated for 3–5 days. Leaves were observed by confocal microscopy (Nikon Eclipse TE2000-U microscope with Nikon Plan 20x/0.40 ∞/0.17 WD; 1.3 and Plan Apo 60x/1.20 WI ∞/0.15–0.18 WD; 0.22 objectives. The confocal microscope included a CL-2000 diode pumped crystal laser (LaserPhysics Inc.), and a LS 300 Kr/Ar laser (Dynamic Laser), a Photometrics CascadeII 512 camera and a QLC-100 spinning disc (VisiTech international)). Metamorph software was used to acquire the images (version 7.7.7.0; Molecular Devices). All fluorescence images were taken as 17 single optical sections and Z-stacked using maximum projections with FIJI software^84^.

### Co-immunoprecipitation (Co-IP)

The *PYL6* cDNA was cloned in a pGPTVII-Bar vector backbone with N-terminal Venus (PYL6-Venus) and MYC2 in a 6xHIS-3XFlag vector (HF-MYC2) both driven by the Ubiquitin 10 promoter. They were transformed into the agrobacterium GV3101 strain by electroporation and infiltrated in *N. benthamiana* as mentioned above. After 4 days, transformed leaves were harvested and frozen in liquid nitrogen. Total protein was extracted by grinding the tissue in extraction buffer (3 mL/g of leaf; 100 mM sodium phosphate pH 8.0, 150 mM NaCl, 5 mM EDTA, 5 mM EGTA, 0.1% TX-100, 1 mM PMSF, 1x phosphatase inhibitors II and III (Sigma) and protease inhibitor cocktail (Roche)). The samples were split and 50 μM ± ABA was added to one sample and the corresponding amount of ethanol to the other samples. Samples were sonicated 2 × 10 seconds on ice and cell debris was spun down by centrifugation. The samples were filtered in a 45 μm filter and the input sample was collected. After equilibrating magnetic beads (Sigma, M2) with the extraction buffer, sample and beads were incubated at 4 °C for 2 hours. Then, the beads were washed with extraction buffer first and then with FLAG to His buffer (100 mM sodium phosphate pH 8, 150 mM NaCl and 0.05% TX-100). Finally, the HF-MYC2 was eluted in FLAG to His buffer + 500 μg/mL of 3x FLAG peptide. The protein samples were separated in a SDS 10% polyacrylamide gel and transferred to a PVDF membrane for Western blots.

### Yeast one hybrid

A 195 bp DNA fragment from the JAZ6 promoter (−975 to −779 42) was cloned in a pLacZi vector backbone with a USER cassette. This fragment contained 2 G-box sequences. The construct was digested with NcoI and integrated into the genome of the yeast strain YM4271. Using the vectors described for the Y2H, this yeast strain was co-transformed either with AD-MYC2 and empty BD vector, or with AD-MYC2 and BD-PYL6. After isolation of transformants in triple dropout media SD-Ura-Leu-Trp, 3 colonies were selected for liquid culture assays using ONPG (Sigma) as substrate (following Clontech Yeast Protocols Handbook instructions). The same procedure was followed for JAZ8 promoter fragments (−237 to −24 42) with the difference that 6 copies of the same 213bp DNA sequence were cloned in tandem in pLacZi.

### Plant growth

Columbia-0 wild type Arabidopsis plants and *pyl6* (SALK_206633C) mutant plants were used. Synchronized seeds were sterilized by incubation in sterilization medium (70% Ethanol, 0.1% SDS and 0.1% bleach) for 15 min followed by 3 washes in 100% Ethanol. After drying, the seeds were sowed on 1% phytoagar, 1% sucrose, ½ strength Murashige and Skoog (MS) medium, MS + 0.5 μM ABA, MS + 10 μM Methyl-Jasmonate or both hormones, MS+ 0.5 μM ABA + 10μM Methyl-Jasmonate. Plates were then stored at 4 °C for >3 days and subsequently transferred to a growth cabinet (16/8 light/dark and 22 °C). Seed germination was measured as radicle emergence during the first 3 days, and cotyledon greening as number of expanded green cotyledons per number of sowed plants at the indicated time points.

### Protein Overexpression and Purification

The MYC2-StrepII fusion was cloned in a USER-modified pGEX-6P-1 vector. Overexpression of recombinant proteins was performed with Rosetta (EMD Millipore) *E. coli* cells after the addition of 0.5 mM of IPTG for 4 hours at room temperature. For the isolation of ABI1, additional 5 mM MgCl_2_ was added to the buffer in which the bacterial pellet was resuspended (buffer W in IBA manual). Also, proteins were eluted in elution buffer supplemented with 20% Glycerol and stored at −80 °C. To assess protein concentrations, several volumes of the eluates were loaded on a gel together with increasing bovine serum albumin (BSA) protein amounts (as concentration ladder). After separating the proteins by SDS-PAGE, the proteins were stained with coomassie brilliant blue R-250, dried between two sheets of cellophane, and then scanned. BSA and recombinant protein band intensities were measured using Fiji. After subtracting the background signal, BSA band signal intensities were used to plot a standard curve. Concentrations of isolated recombinant proteins were then calculated based on the equation resulting from the linear regression of the BSA standard curve.

## Additional Information

**Accession Numbers:** PYL6 (At2g40330), PYL5 (AT5G05440), PYL4 (At2g38310), ABI1 (AT4G26080), MYC2 (Atg32640), MYC3 (At5g46760), JAZ6 (At1g72450), JAZ8 (At1g30135).

**How to cite this article**: Aleman, F. *et al*. An ABA-increased interaction of the PYL6 ABA receptor with MYC2 Transcription Factor: A putative link of ABA and JA signaling. *Sci. Rep*. **6**, 28941; doi: 10.1038/srep28941 (2016).

## Supplementary Material

Supplementary Information
