# Author Correction: An ABA-increased interaction of the PYL6 ABA receptor with MYC2 Transcription Factor: A putative link of ABA and JA signalling

**DOI:** 10.1038/s41598-025-33609-z

**Published:** 2026-01-08

**Authors:** Fernando Aleman, Junshi Yazaki, Melissa Lee, Yohei Takahashi, Alice Y. Kim, Zixing Li, Toshinori Kinoshita, Joseph R. Ecker, Julian I. Schroeder

**Affiliations:** 1 Division of Biological Sciences, Cell and Developmental Biology Section, University of California San Diego, La Jolla, California USA; 2 Plant Biology Laboratory, Genomic Analysis Laboratory, The Salk Institute for Biological Studies, La Jolla, California 92037 USA; 3 Division of Biological Science, Graduate School of Science, Nagoya University, Nagoya, 464-8602 Japan; 4 Institute of Transformative Bio-Molecules (WPI-ITbM), Nagoya University, Nagoya, 464-8602 Japan; 5 Howard Hughes Medical Institute, The Salk Institute for Biological Studies, La Jolla, California 92037 USA; 6 Present Address: The Scripps Research Institute, 10550 North Torrey Pines Road, La Jolla, California USA; 7 Present Address: RIKEN Center for Integrative Medical Sciences, 1-7-22 Suehiro-cho Tsurumiku, Yokohama, Kanagawa Japan

Correction to: *Scientific Reports* 10.1038/srep28941, published online 30 June 2016

The original version of this Article contains two errors - in Figure 4 and in the Additional information that occurred during manuscript preparation.

In Figure 4, as a result of errors during figure assembly, an incorrect duplicate image of *pyl6* mutant seedlings is shown for wildtype control seedlings in the presence of 10 µM JA in Figure 4B. The corrected Figure 4 and accompanying legend appear below.

**Fig. 4:**
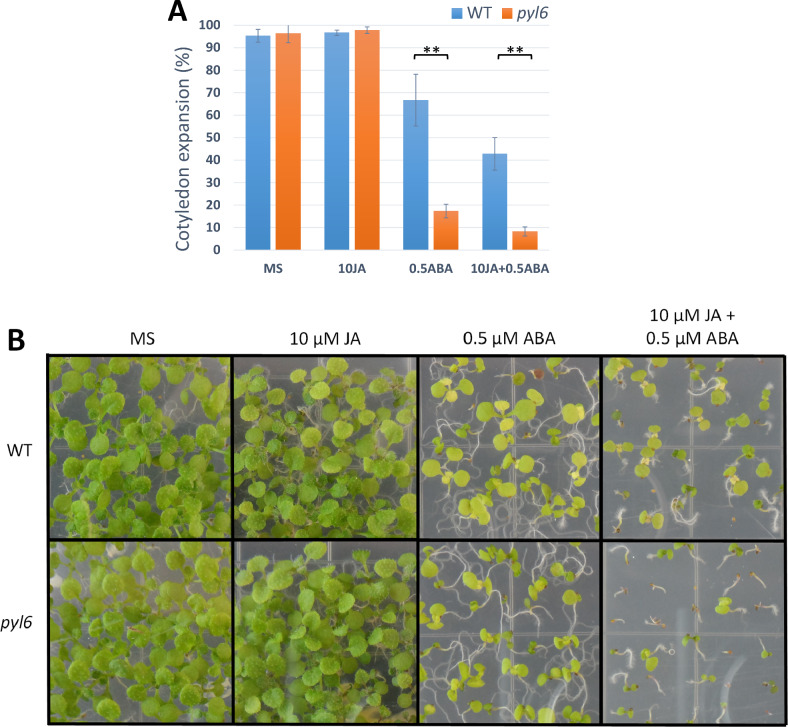
*pyl6* seedlings are more sensitive to ABA and to the combination of ABA and JA than WT (Col-0). (**A**) 5 days after sowing expanded and green cotyledons were quantified per number of plants analyzed (%). Data are represented as mean ± SD and **Indicates *p*-value < 0.01 after a two-tailed t-test. Images of the seedlings at this stage can be found in Supplementary Figure S4. (**B**) In 11 day-old seedlings, the synergistic action of JA over the ABA effect was enhanced in *pyl6* mutants compared to WT. Right panels show 0.5 μM ABA plus 10 μM Me-JA.

In the Additional information, the Accession number for MYC2 is incorrectly noted as Atg32640 and should be At1g32640.

In the Additional information,

“Accession Numbers: PYL6 (At2g40330), PYL5 (AT5G05440), PYL4 (At2g38310), ABI1 (AT4G26080), MYC2 (Atg32640), MYC3 (At5g46760), JAZ6 (At1g72450), JAZ8 (At1g30135).”

should read:

“Accession Numbers: PYL6 (At2g40330), PYL5 (AT5G05440), PYL4 (At2g38310), ABI1 (AT4G26080), MYC2 (**At1g32640**), MYC3 (At5g46760), JAZ6 (At1g72450), JAZ8 (At1g30135)”.

