# Supplementary material for: An ABA-increased interaction of the PYL6 ABA receptor with MYC2 Transcription Factor: A putative link of ABA and JA signaling
Source: Sci Rep. 2016 Jun 30;6:28941. doi: 10.1038/srep28941 (PMC4928087; doi:10.1038/srep28941)
Supplement: Supplementary Information [file srep28941-s1.pdf]

An ABA-increased interaction of the PYL6 ABA receptor with MYC2 Transcription Factor: A putative link of ABA and JA signaling.

Fernando Aleman[1¶], Junshi Yazaki[2Ψ], Melissa Lee[1], Yohei Takahashi[1,3], Alice Y. Kim[2], Zixing Li[1], Toshinori Kinoshita[3,4], Joseph R. Ecker[2,5] and Julian I. Schroeder[1*].

1 Division of Biological Sciences, Cell and Developmental Biology Section, University of California San Diego, La Jolla, California, USA.

2 Plant Biology Laboratory, Genomic Analysis Laboratory, The Salk Institute for Biological Studies, La Jolla, California, 92037 USA.

3 Division of Biological Science, Graduate School of Science, and **4** Institute of Transformative Bio-Molecules (WPI-ITbM), Nagoya University, Nagoya 464-8602, Japan.

5 Howard Hughes Medical Institute, The Salk Institute for Biological Studies, La Jolla, California, 92037 USA.

Ψ Present address: RIKEN Center for Integrative Medical Sciences, 1-7-22 Suehiro-cho Tsurumiku, Yokohama, Kanagawa, Japan.

¶ Present address: The Scripps Research Institute, 10550 North Torrey Pines Road, La Jolla, California, USA (faleman@scripps.edu).

*Correspondence should be addressed to Julian I. Schroeder, Division of Biological Sciences, Cell and Developmental Biology Section, University of California San Diego, 9500 Gilman Drive, La Jolla, CA, 92093-0116, USA (jischroeder@ucsd.edu).

Figure S1: Negative controls with the empty vector (BD).

Figure S2: *pyl6* mutant protein does not interact with MYC2 and interacts with ABI1 only in the presence of ABA.

Figure S3: No seed germination difference in *pyl6* mutant plants compared to control.

Figure S4: *pyl6* mutant plants show a reduced cotyledon emergence phenotype in the presence of 0.5µM ABA after 5days of growth.

Figure S5: MYC2 does not impaire PYL6-induced inhibition of ABI1 protein phosphatase activity.

Figure S6: Expression levels of JAZ6 and JAZ8 in Col-0 and *pyl6* mutants under control conditions and treated with 0.5 µM ABA.

Table S1: Primers used.

Supplementary:

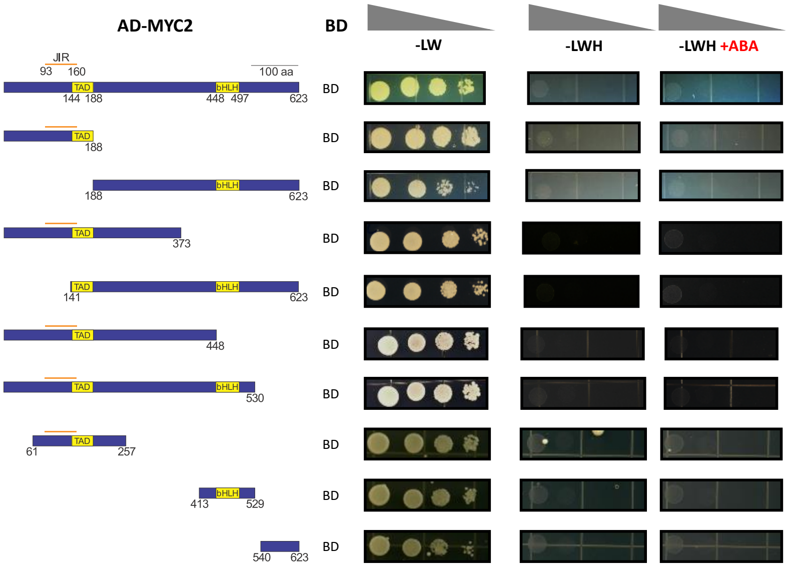

Figure S1: Negative controls with the empty vector (BD).

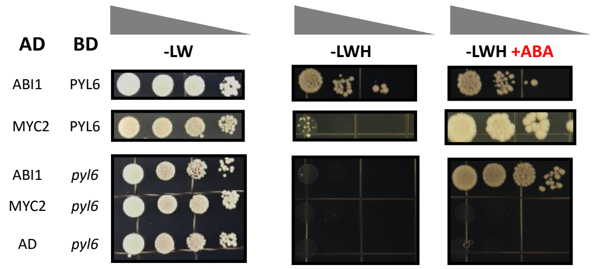

Figure S2: *pyl6* mutant protein does not interact with MYC2 and interacts with ABI1 only in the presence of ABA in yeast 2 hybrid assays. In the first row, WT PYL6 protein was analyzed with ABI1 as a positive control. The T-DNA insertion disrupts the last α-helix domain in which two non-polar residues have been reported to be important for the binding of ABA in the case of PYL9 (Nakagawa et al. 2014).

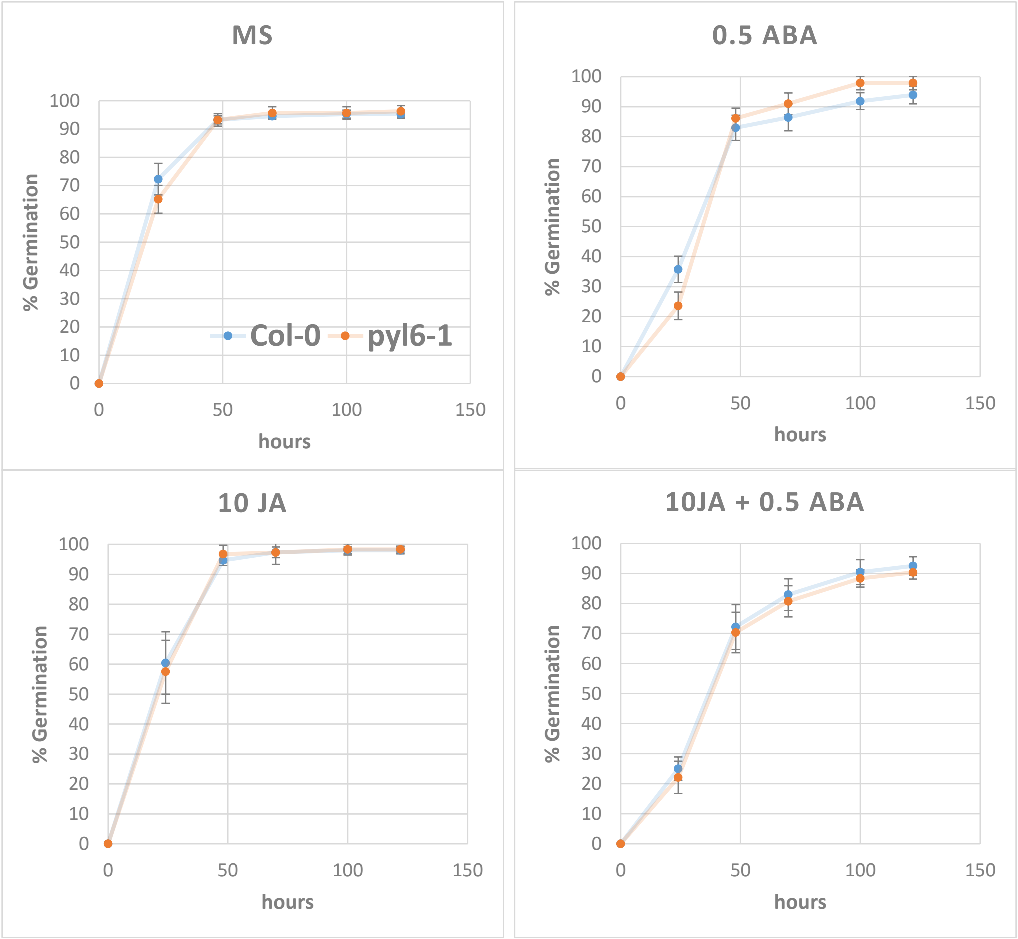

Figure S3: No seed germination difference in *pyl6* mutant plants compared to control. Col-0 (WT) and *pyl6* mutant seeds were sown on 1% sucrose MS media and MS supplemented with 0.5 µM ABA, 10 µM Me-JA, and 0.5 µM ABA + 10 µM Me-JA. No differences were found on the media with sucrose.

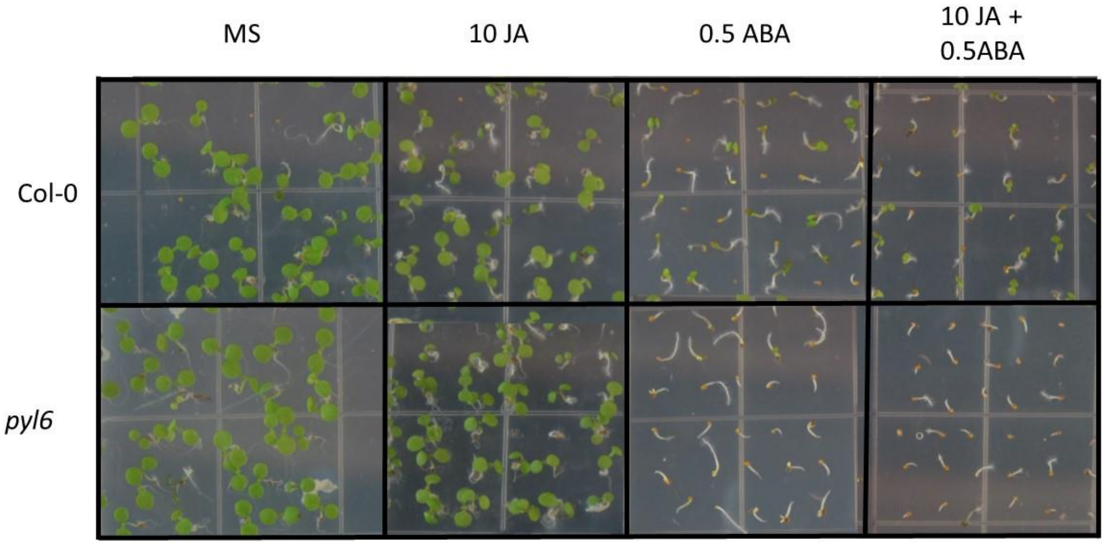

Figure S4: *pyl6* mutant plants show a reduced cotyledon emergence phenotype in the presence of 0.5µM ABA after 5days of growth. The presence of 10µM JA plus 0.5µM ABA further increased this phenotype.

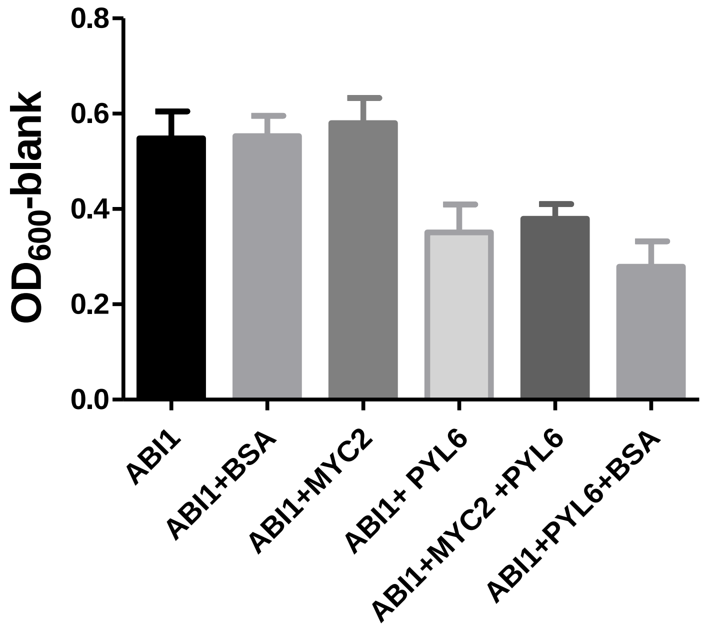

Figure S5: MYC2 does not impaire PYL6-induced inhibition of ABI1 protein phosphatase activity. The first 3 columns show ABI1 protein phosphatase activity alone, with BSA and with MYC2. The last 3 columns show ABI1 activity with PYL6, and with the addition of either MYC2 or BSA. 10 µM ABA was added for all conditions.

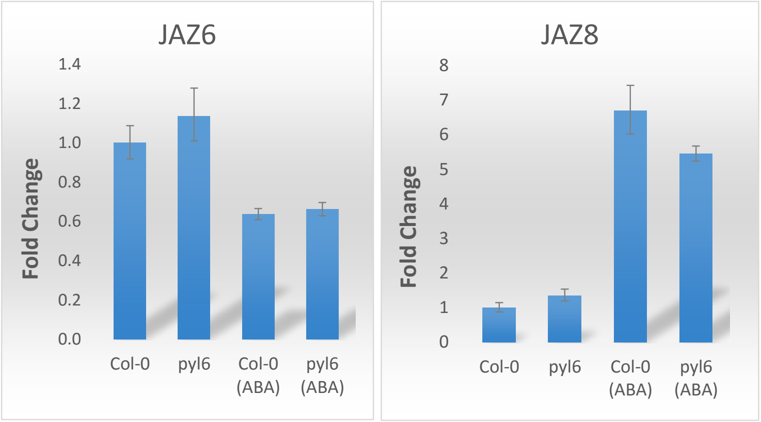

Figure S6: Expression level of JAZ6 and JAZ8 in Col-0 and *pyl6* mutants under control conditions and treated with 0.5 µM ABA. Col-0 and *pyl6* mutants seeds were sown in ½ MS control media or ½ MS supplemented with 0.5 µM ABA. qPCR experiments were performed with 11 day-old seedlings and the expression profile normlized to Col-0 in ½ MS media using the $\Delta\Delta C_t$ method.

Sequences	AGI	Notes
GGCTTAAUatgccaacgtcgatacagtttc	AT2G40330	PYL6-ATG user cloning
GGTTTAAUTTACGAGAATTTAGAAGTGTTC	AT2G40330	PYL6-STOP user cloning
GGTTTAAUTCAATTTGCCAGCGATTGCAAG	AT2G40330	PYL6 c-ter delet Y2H (without 11 last aa with STOP)
GGCTTAAUatgactgattaccggctacaac	AT1G32640	MYC2 user ATG
GGTTTAAUTTAACCGATTTTTGAAATCAA	AT1G32640	MYC2 user STOP
GGCTTAAUatgaggtcaccggtgcaactcc	AT5G05440	PYL5 user ATG
GGTTTAAUTTATTGCCGGTTGGTACTTCGAG	AT5G05440	PYL5 user STOP rev
GGCTTAAUatgaacggcacaacatcatcaatc	AT5G46760	MYC3 user ATG
GGTTTAAUTCAATAGTTTTCTCCGACTTTCG	AT5G46760	MYC3 user STOP rev
GGTTTAAUccCGAGAATTTAGAAGTGTTCTC	AT2G40330	PYL6 Reverse cc W/out STOP codon user
GGTTTAAUtcaTGCAAACGCTTTACCAGCTAAT	AT1G32640	MYC2 user 188 reverse
GGTTTAAUacgggtaacgcggtttgggtttc	AT1G32640	MYC2 user 188 forward
GGCTTAAUGAGCTTAACTCGTTGATCTCC	AT1G32640	MYC2 from Glu141 user forward
GGTTTAAUtcaACCTTCATCGCCGAAGTTTAATA	AT1G32640	MYC2 from Gly373 user reverse +STOP
GGTTTAAUTTATTCTCTACCGTTTGCTGGCTT	AT1G32640	MYC2 448aa reverse +STOP
GGTTTAAUTTAACTAGCACTCGCTTTTCTTCCG	AT1G32640	MYC2 530aa reverse +STOP
GGCTTAAUgcgggatttaatcaagagactc	AT1G32640	MYC2 Ala61 Fw user
GGTTTAAUTTATCCGCCGTCGAAATTGAAAAG	AT1G32640	MYC2 Gly257 Rv user (+STOP)
GGCTTAAUaccgccggagaatcagatcac	AT1G32640	MYC2 Thr413 Fw user
GGTTTAAUTTAAGCACTCGCTTTTCTTCCGGC	AT1G32640	MYC Ala529 Rv user (+STOP)
GGCTTAAUTCGATTAAACCGGTGGGGATGG	AT1G32640	MYC Ser540 Fw user
GGCTTAAUtgcatgaaataaggttggtaccg	AT1G72450	JAZ6 promoter fw
GGTTTAAUTCGGCTGCGATGGCTAATAC	AT1G72450	JAZ6 promoter Rv
GGCTTAAUacaaatccaaaaggaccaa	At1g30135	JAZ8 user promoter forward
GGTTTAAUGTATATGGATTGAGATTATGTTGAT	At1g30135	JAZ8 user promoter reverse
ACTTGGTAuGTATATGGATTGAGATTATGTTGAT	At1g30135	JAZ8 promoter F1 reverse
ATACCAAGuacaaatccaaaaggaccaa	At1g30135	JAZ8 promoter F2 forward
ATATATAuGTATATGGATTGAGATTATGTTGAT	At1g30135	JAZ8 promoter F2 reverse
ATATATAuacaaatccaaaaggaccaa	At1g30135	JAZ8 promoter F3 forward
AGTGAGTACAAuGTATATGGATTGAGATTATGTTGAT	At1g30135	JAZ8 reverse fragment A
attgtactcacUacaaatccaaaaggaccaa	At1g30135	JAZ8 forward fragment B
ttctcagtgcttctccgtcgtg	AT2G40330	PYL6 Forward for qPCR
ATCGACCAGACTGTGGAAACCG	AT2G40330	PYL6 Reverse for qPCR
ggtcggatcctccaaacaagtc	At1g30135	JAZ8 Forward qPCR
CGTCGTGAATGGTACGGTGAAG	At1g30135	JAZ8 Reverse qPCR
tcccgatcttaacgagccaacg	AT1G72450	JAZ6 Forward qPCR
ACCTGATGTTGCTGCCCAGTTTC	AT1G72450	JAZ6 Reverse qPCR

Supporting Table: Table S1: Primers used.

Nakagawa M, Kagiyama M, Shibata N, Hirano Y, Hakoshima T (2014) Mechanism of high-affinity abscisic acid binding to PYL9/RCAR1. Genes to Cells 19 (5):386-404. doi:10.1111/gtc.12140
